# Targeting DNA Repair Response Promotes Immunotherapy in Ovarian Cancer: Rationale and Clinical Application

**DOI:** 10.3389/fimmu.2021.661115

**Published:** 2021-10-12

**Authors:** Hongyu Xie, Wenjie Wang, Wencai Qi, Weilin Jin, Bairong Xia

**Affiliations:** ^1^ Clinical Research Center, Women’s Hospital School of Medicine Zhejiang University, Hangzhou, China; ^2^ Department of Gynecology Oncology, Harbin Medical University Cancer Hospital, Harbin, China; ^3^ Department of Epidemiology and Biostatistics, School of Public Health, Harbin Medical University, Harbin, China; ^4^ Department of Gynecology Oncology, Division of Life Sciences and Medicine, The First Affiliated Hospital of University of Science and Technology, Hefei, China; ^5^ Institute of Cancer Neuroscience, Medical Frontier Innovation Research Center, The First Hospital of Lanzhou University, The First Clinical Medical College of Lanzhou University, Lanzhou, China

**Keywords:** ovarian cancer, DNA damage response, PARP inhibitors, combination therapy, immune checkpoint inhibitors (ICI)

## Abstract

Immune checkpoint inhibitors (ICI) have emerged as a powerful oncologic treatment modality for patients with different solid tumors. Unfortunately, the efficacy of ICI monotherapy in ovarian cancer is limited, and combination therapy provides a new opportunity for immunotherapy in ovarian cancer. DNA damage repair (DDR) pathways play central roles in the maintenance of genomic integrity and promote the progression of cancer. A deficiency in DDR genes can cause different degrees of DNA damage that enhance local antigen release, resulting in systemic antitumor immune responses. Thus, the combination of DDR inhibitors with ICI represents an attractive therapeutic strategy with the potential to improve the clinical outcomes of patients with ovarian cancer. In this review, we provide an overview of the interconnectivity between DDR pathway deficiency and immune response, summarize available clinical trials on the combination therapy in ovarian cancer, and discuss the potential predictive biomarkers that can be utilized to guide the use of combination therapy.

## Background

Immune checkpoint inhibitors (ICI), such as CTLA-1, PD-1, and PD-L1, have emerged as a powerful oncologic treatment modality and have become a standard treatment for patients with different tumor types (1, 2). Unfortunately, the results of several studies on monotherapy ICI agents in epithelial ovarian cancer (EOC) are disappointing (3, 4). Different combinatorial therapeutic strategies to enhance tumor immunogenicity are needed to improve the efficacy of ICI therapy in ovarian cancer. The data suggest that DNA damage repair (DDR) deficiency promotes local antigen release, resulting in systemic antitumor immune responses (5). As such, combining ICI agents with DDR-targeting agents provides a new opportunity in the treatment of ovarian cancer.

DNA damage constantly occurs in cells under the threat of both exogenous and endogenous stressors. Under these conditions, cells initiate a series of DNA damage responses to maintain genome integrity. DNA damage commonly falls into single-strand breaks (SSBs) and double-strand breaks (DSBs), and the DDR pathways are used to repair them through damage recognition and DNA repair depending on the specific type of damage detected. In normal cells, if the DNA damage is too large that it exceeds the capacity of DDR, then an apoptosis program is activated to eliminate unrepaired DNA damage. In cancer cells, the damaged DNA, which may be due to endogenous physiological factors (*e*.*g*., aldehydes and reactive oxygen species) or external physical and chemical therapeutic agents (*e*.*g*., ionizing radiation and platinum drugs), is often complex and exceeds the repair capacity of DNA damage responses. Moreover, cancer cells have the characteristics of high division rate and rapid accumulation of related aberrations, which lead to genome instability (6). Inhibition of DDR is an effective therapeutic strategy for cancers. For ovarian cancer, mutations in the homologous recombination (HR) repair genes BRCA1 and BRCA2 are the most common alterations and inhibitions of the DDR pathway protein poly(ADP ribose) polymerase (PARP). These mutations are an attractive synthetic lethal target for therapy with the greatest efficacy observed. A phase III study on patients with platinum-sensitive recurrent ovarian cancer taking PARP inhibitors as maintenance therapy found that these patients had longer progression-free survival compared with the placebo group, independent of the BRCA1/2 mutation status or other HR repair gene status (7). A study on breast cancer cell lines and animal models revealed that PARP inhibitors upregulated PD-L1 by inactivating GSK3β, and the combination of PARP inhibitors and anti-PD-L1 demonstrated better therapeutic benefit than each treatment alone (8). The combination of PARP inhibitors and ICI therapy is being tested in several clinical trials in ovarian cancer currently. A better understanding of the interconnectivity between PARP inhibitor and immune responses would facilitate efforts in the development of single and combination of agents.

In this review, we discuss in detail the molecular mechanisms by which PARP inhibitor treatment induces immune responses, summarize available clinical data on combination therapy in ovarian cancer, and explore the potential predictive biomarkers that are utilized to guide the use of combination therapy

## DNA Damage Repair Pathways in Ovarian Cancer

Over 450 proteins have been identified to be involved in DDR pathways. These proteins are involved in various DDR pathways according to their mechanisms of action and functions to different types of DNA damage (9). Mismatch repair (MMR) is one of the most widely studied DDR pathways. The principle of MMR is that, in the process of replication, base mismatches can distort the helical structure of a DNA, which will lead to the excision of the mismatched DNA and then replacement of the damaged site with newly synthesized DNA (10). The MMR pathway can identify abnormalities in DNA strands and repair defects. The main proteins involved in these processes are MSH2, MSH3, and MSH6 and MLH1, MLH3, PMS1, PMS2, and PMS3, respectively (11, 12). About 29% of ovarian cancer cases have been found to lose the function in any of the MMR pathway proteins (13). Base excision repair (BER) is the major repair route for endogenous SSBs. This pathway removes damaged bases from the double helix and excises the damaged section from the DNA structure (14). In ovarian cancers, overexpression of the BER pathway proteins FEN1 and XRCC1 are reportedly linked to high clinical stage and poor survival (15, 16). Nucleotide excision repair (NER), the removal of large DNA lesions, is specific to single-strand DDR that causes structural distortions within the DNA double-helix (17). The NER pathway proteins are key factors involved in response to treatment and prognosis in ovarian cancer, among which the most important are XPA-G, XRCC1-DNA ligase, RPA, polymerase epsilon, RAD23A and B, CAS and CSB, and ERCC1 (18, 19). HR is an accurate and error-free pathway utilized to detect and repair DSBs. This process is mainly confined to the S and G2 phases of the cell cycle. Nucleotides are removed from both upstream and downstream at the damaged site, and new DNA is synthesized using the homologous sister chromatid as a template (20). The HR pathway includes various molecules, such as BRCA1, BRCA2, and XRCC2/3 (21, 22). In high-grade serous ovarian cancer, BRCA1/BRCA2 gene mutations are important players in the HR pathway and account for 20% of patients with ovarian cancer with BRCA1/BRCA2 somatic or germline mutations (23). Additionally, mutations in other genes involved in the HR pathway are seen in ovarian cancers (24). Non-homologous end joining (NHEJ), an independent additional repair pathway, functions throughout the cell cycle to repair DSBs (25). NHEJ does not require a homologous template, unlike the HR pathway, which is mediated by joining the ends of broken DNA strands together and therefore is prone to high rates of DNA deletion and mutation. NHEJ pathway proteins mainly include DNA-dependent protein kinase catalytic subunit, Ku70, Ku80, Artemis, XRCC4, XLF/Cernummos, and ligase IV (26, 27). Defects in NHEJ in ovarian cancer are more likely to be resistant to treatment with PARP inhibitor (28). Alternative endjoining (A-EJ) pathways are utilized to perform DSB repair. Based on the number of complementary DNA sequences used to align the ends of DNA, A-EJ includes three distinct pathways, namely, single-strand annealing (SSA), microhomology-mediated end joining (MMEJ), and end joining (EJ) pathways, which is similar to NHEJ in that it does not use a homologous template in the process of joining the ends of DNA. A-EJ is an alternative approach to DSB repair and a potential therapeutic for HR- and NHEJ-deficient cancers (29). In addition, there are many other DSB repair mechanisms. Galanty et al. demonstrated that PIAS1 and PIAS4 promoted DSB repair and conferred ionizing radiation resistance (30). Mirman et al. revealed that CTC1-STN1-TEN1-Polα-mediated fill-in helps to control the repair of DSB by 53BP1, RIF1, and shieldin (31). PI3K-related ATM kinase can trigger the chromatin domains decorated with phosphorylated histone H2AX and form the DDR foci (32, 33). Furthermore, interstrand crosslink repair (ICL repair) for repair of ICL injury is formed by alkylating agents (34). Fanconi anemia pathway is thought to involve the ICL repair, and proteins implicated in this pathway include FANCA, FANCB, FANCC, *etc.* (35). Mutations in one of the FANC gene lead to severe sensitivity to ICL agents and genomic instability (36). In addition, proteins with other biochemical functions also play an important role in the repair of ICLs, such as XP, CS, COFS, and TTD. A study on hereditary breast cancer/ovarian cancer revealed that the mutations in BRCA1, BRCA2, RAD51, PALB2, and PRIP1 are associated with the ICL repair pathways (34).

Compared with SSBs, DSBs are more lethal to cells, and rapid countermeasures are needed to ensure cell survival. Therefore, DDR pathways provide vulnerabilities to kill cancer cells without affecting the normal cells that target these specific pathways, thereby increasing replication stress and thus the frequency of DSBs (37). Cancer cells with defects in DDR exhibit a hypersensitivity to drugs targeting DDR. The use of PARP inhibitors for the treatment of HR-defective ovarian cancer is a successful example. PARP inhibitors inhibit the catalytic activity of PARP protein and block the DDR mechanisms dependent on it. Moreover, PARP inhibitors can trap PARP protein in the DNA and obstruct the replication fork progression. Both mechanisms can cause fatal DNA damage to HR-deficient tumor cells (38). A growing number of research suggest that DDR defects can promote the response and sensitivity to immunotherapy (39, 40).

## Links Between PARP Inhibitors and Immune Responses in Ovarian Cancer

### Neoantigen Dependence

**Figure 1:**
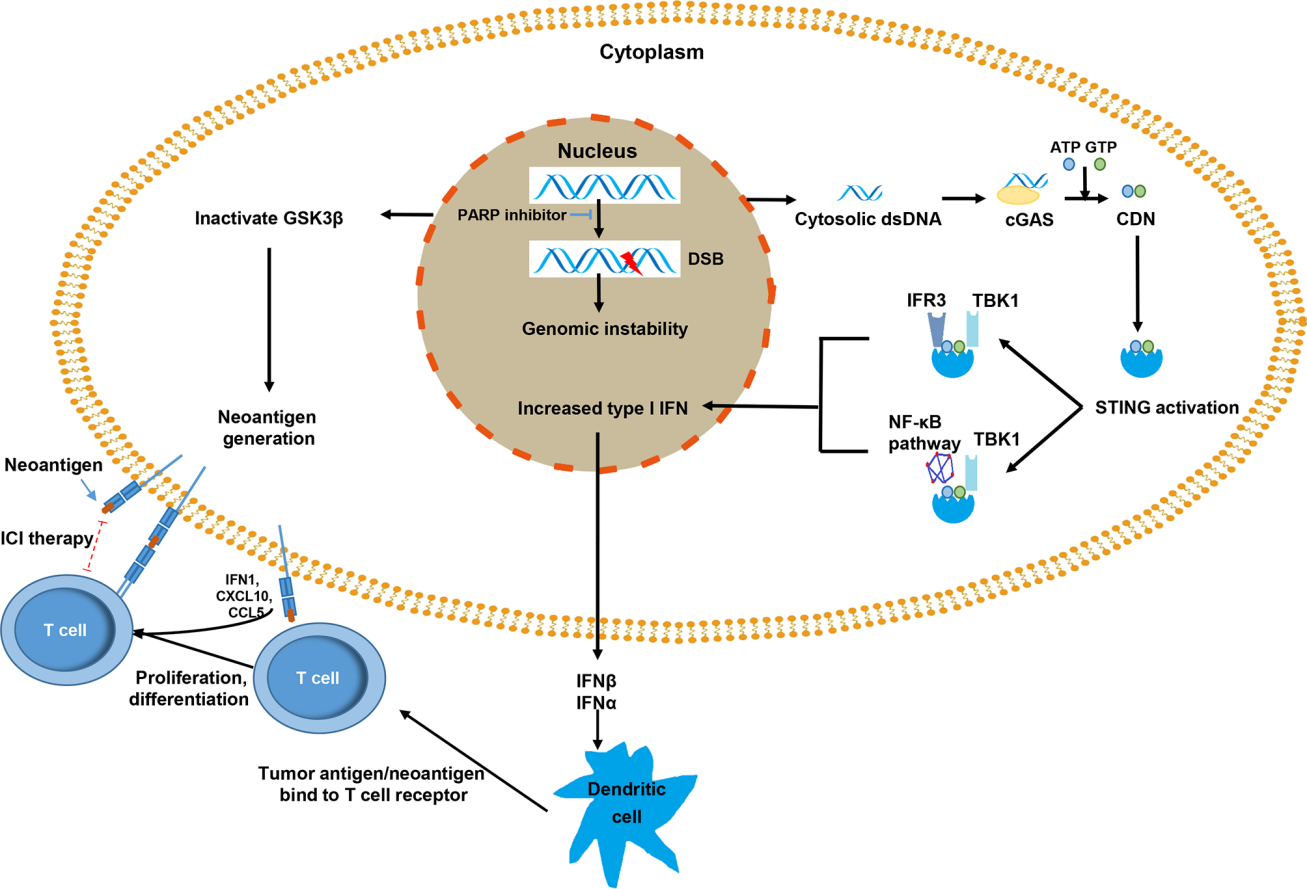
The mechanisms by which poly(ADP ribose) polymerase (PARP) inhibitors promote immune response against tumor cells. The PARP inhibitor inactivates GSK3β, which can lead to increased PD-L1 expression in tumor cells. The release of dsDNA and the activation of the cGAS–STING pathway results in the increased expression of type I IFN *via* the TBK1-IRF3 and TBK1-NF-κB pathways. Type I IFN activates immune effector cells by promoting the presentation of tumor dendritic cell antigen/neoantigen, and the major histocompatibility complex of a dendritic cell binds to T cell receptors and activates T cells. The activated T cells infiltrate the tumors and recognize tumor antigens that presented on the tumor cell surface. PD-L1 expression, as an immune escape signal, can be targeted with immune checkpoint inhibitor therapy (red dotted line).

Defects in the DDR pathway are often associated with an increased tumor mutation burden (TMB) (41). However, a study on Lynch syndrome reported that a deficiency in MMR can also lead to a low TMB and found that the discordance of tumor with deficiency in MMR and TMB may make it resistant to immunotherapy (39). TMB is considered an alternative for neoantigen burden (42). High neoantigens stimulate the increase of tumor-infiltrating lymphocytes (TILs), and accumulated TILs can be counterbalanced by the overexpression of immune checkpoint regulators, such as PD-L1 and PD-1 (43, 44). In ovarian cancer, BRCA1/2-mutated tumors with a higher level of neoantigens than those without alterations in HR genes and BRCA1/2 defects are associated with an increase of PD-L1 expression and T-cell infiltration (45, 46). However, the immunogenicity of BRCA1/2 mutation-associated ovarian cancers does not exhibit improved responses to ICI, most likely because of the overall low TMB, and carry a limited predictive value in ovarian cancer (47). PARP inhibitor-mediated catastrophic DNA damage often heralds the therapeutic response of ICI treatment independently of BRCA1/2 mutations. However, no evidence has demonstrated yet whether PARP inhibitors might increase TMB to improve the efficacy of ICI treatment in cancer cells. PARP inhibitors could inactivate GSK3β, which increased PARP inhibitor-mediated PD-L1 upregulation and enhanced cancer-associated immunosuppression. Anti-PD-L1 could potentiate the anti-tumor efficacy of PARP inhibitors compared with each agent alone significantly (8) (**Figure 1**). In fact, although neoantigens and the immunogenicity of tumors correlated with improved outcomes to ICI, no threshold has been established that clearly discriminate responders and nonresponders to ICI therapy. Numerous studies have shown that low TMB can also be sensitive to ICI, and increased tumor neoantigen burden (TNB) does not correlate with T cell inflammation in some human tumors (48, 49). DDR deficiency in driving ICI treatment with non-neoantigen-dependent mechanisms has been proposed (50, 51).

### Non-Neoantigen-Based Mechanisms

Failure of DDR of cancer cells, genomic instability, and incomplete DNA repair repertoire usually lead to chromosome mis-segregation during cell division (52), which are wrapped by primitive nuclear membrane and further forms micronuclei (53). The membrane of micronuclei is easy to rupture and double-strand DNA (dsDNA) is exposed in the cytoplasm (54, 55). Cytosolic dsDNA stimulates the activation of cyclic GMP-AMP synthase (cGAS) and subsequently catalyzes the generation of cyclic dinucleotide (CDN) (56). CDN is a second messenger, a unique phosphodiester linkage that uses ATP and GTP (56, 57), which promotes the activation of the stimulator of interferon genes (STING). Activated STING mainly recruits TBK1 to further phosphorylate transcription IRF3 and upregulates the expression of inflammatory cytokines and type I IFN (58). Type I IFN is essential for dendritic cells (DCs) and CD8^+^ T cells in their antitumor immune responses (59, 60). Furthermore, the STING–TBK1 association phosphorylates IκB kinase, leading to the noncanonical activation of the NF-κB pathway. In turn, this pathway cooperates with the TBK1–IRF3 pathway to induce the expression of type I IFN (55). Type I IFN has a substantial influence on systemic immune response and promotes the maturation, migration, and activation of immune cells, especially DCs, natural killer cells, and T cells (**Figure 1**) (61, 62).

DDR defects can dramatically impact the microenvironment. The cytosolic DNA-mediated cGAS–STING pathway promotes the reshaping of the immune environment, thus making tumor cells more sensitive to be killed by immune cells (63, 64). cGAS interacts with PARP1 and impedes the formation of the PARP1-Timeless complex *via* poly(ADP-ribose), which suppresses HR (65). cGAS can also suppress HR by impeding RAD51-mediated DNA strand invasion (66). All these processes can lead to DDR deficiency. Owing to the activation of the cGAS–STING pathway, DDR-deficient tumors can increase immune infiltration and elevate the level of PD-L1 expression by PARP inhibitors through the inactivation of GSK3β activity (8) and by BRCA2 and ku70/80 deficiency in an IFNα- and CHEK1-dependent manner (67, 68). In addition, cancer-derived DNA can stimulate the cGAS–STING-type I IFN pathway, which subsequently enhances the recruitment and activation of T cells (61) and weakens the immunosuppressive function of Treg by downregulating the level of cyclic AMP (69).

PARP inhibitors lead to the failure of DDR and promote the accumulation of cytosolic dsDNA, which activates the cGAS–STING pathway, thereby stimulating the production of type I IFN to induce antitumor immunity and enhancing the recruitment and infiltration of T cells into tumors (70). A study on the loss of BRCA1 and p53 and overexpression of c-Myc in high-grade serous ovarian cancer model of syngeneic genetically engineered mouse showed that PARP inhibitors induced the activation of the STING pathway, accompanied by an increased expression of IFNβ, PD-L1, and CXCL10 (71). In a HR-proficient ID8 model, PARP inhibitor talazoparib induces STING activation, increases the expression of CCL5, CXCL10, and PD-L1, and exhibits synergistic activity with an anti-PD-L1 antibody (72). The increased levels of chemokines induce the activation of cytotoxic CD8^+^ T cell (70).

DDR deficiency may also increase the sensitivity of tumor to ICI by activating other signaling pathways (73)—for example, in pancreatic tumor, inhibition of ATM increases the expression of tumor type I IFN through a SRC- and TBK1-dependent manner. Moreover, ATM silencing increases PD-L1 expression and increases the sensitivity to anti-PD-L1 therapy. In preclinical models, ATM and ATR have been shown to upregulate NKG2DL, which binds the NKG2D receptor, triggering degranulation and cytokine production and contributing to inflammation and NK-mediated cytotoxicity. Defects of the MMR pathway lead to the accumulation of mismatch errors, resulting in microsatellite instability (MSI) and tumorigenesis (74). Tumors with MSI are associated with T cell infiltration and high neoantigen load (75). Chan et al. found that the deficiency of RecQ DNA helicase WRN can cause DDBs, apoptosis, and cell cycle arrest in MSI tumor cells, indicating that WRN is a lethal target for MSI tumor synthesis and can improve the efficacy of ICI therapy (76). Together these results underscore that tumor with underlying DNA repair defects may better respond to ICIs, and targeting DDR is an effective strategy for increasing the efficacy of ICI for cancer therapy (77).

### Tumor Immune Escape of DDR-Deficient Tumor

Despite that DNA damage can promote immune activation, immunotherapeutic agents produce strong and durable responses in only a subgroup of DDR-deficient patients because a tumor with a DDR deficiency can eventually escape immune control and grow unchecked. One of the reasons that DNA damage fails to be eliminated is DDR defects, but at a low level that is not fatal to tumors. This failure could drive inflammatory signaling, stimulate continued infiltration by innate immune cells, and promote the release of free radicals. This series of processes leads to further DNA damage and promotes the transformation from a Th1-skewed immunity to chronic inflammation and immunosuppression in the immune microenvironment, both of which promote cancer progression and immune escape (78, 79). Breaking through a self-sustaining cycle of DNA damage and chronic inflammation is challenging by using any single therapeutic approach. Nevertheless, a combination of drugs, such as PARP inhibitors combined with ICI agents, may offer opportunities for treatment.

## Combination of PARP Inhibitors With Immunotherapies in Ovarian Cancer

The interest in combining immunotherapy with PARP inhibitor in ovarian cancer is growing, owing to PARP inhibitors with the ability of synthetic lethality in cancer cells and their important roles in enhancing the efficiency of immunotherapy. Preclinical studies revealed that PD-L1 blockade augments antitumor effects when given with a PARP inhibitor. Furthermore, PD-L1 blockade prolongs the survival and reduction in tumor growth compared with either agent alone in murine models (71, 73). On the basis of these encouraging preclinical results, multiple clinical studies are recently performed to investigate the clinical activity of PARP inhibitors in combination with immunotherapy for ovarian cancer. These studies were mainly divided into four indications (1): first-line maintenance treatment [*i*.*e*., FIRST trial (NCT03602859), JAVELIN Ovarian PARP 100 (NCT03642132), ATHENA (NCT03522246), DUO-O (NCT03737643), and KEYLYNK-001 (NCT03740165)] (2), platinum-sensitive relapse treatment [*i*.*e*., MEDIOLA (NCT02734004), JAVELIN PARP Medley trial (NCT03330405), NCT04034927, NCT03806049, NCT03695380, and NCT03101280] (3), platinum-resistant relapse treatment [*i*.*e*., TOPACOP/Keynote-162 (NCT02657889), OPAL (NCT03574779), MOONS TONE (NCT03955471)], and (4) independent of the platinum status [*i*.*e*., NCT02571725, NCT02953457, ROCSAN (NCT03651206), ANITA (NCT03598270), NCT02484404, NCT02873962, NCT02485990, ARIES (NCT03824704), and GUIDE2REPAI (NCT04169841)].

Five phase III clinical trials on maintenance setting are ongoing. The FIRST trial was designed to evaluate platinum-based therapy with TSR-042, followed by TSR-042 and niraparib maintenance therapy *versus* standard platinum-based treatment, followed by maintenance niraparib or placebo in advanced ovarian cancer (80). JAVELIN Ovarian PARP 100 was designed to assess the efficacy and safety of avelumab in combination with chemotherapy, followed by avelumab in combination with talazoparib as maintenance therapy *versus* chemotherapy, followed by single-agent talazoparib maintenance or chemotherapy in combination with bevacizumab, followed by bevacizumab maintenance therapy (81). ATHENA is a four-arm study being undertaken to evaluate the efficacy of frontline platinum-based treatment, followed by rucaparib and nivolumab as maintenance treatment in a patient with newly diagnosed ovarian cancer (82). DUO-O evaluated the efficacy and safety of platinum-based chemotherapy in combination with durvalumab and beveacizumab, followed by durvalumab and bevacizumab or durvalumab, bevacizumab, and olaparib as maintenance treatment in patients with newly diagnosed advanced ovarian cancer (83). Finally, KEYLYNK-001 assesses the efficacy and safety of chemotherapy with or without pembrolizumab, followed by olaparib maintenance in patients with EOC (84). Unlike these studies, KEYLYNK-001 regards immunotherapy as a therapeutic agent but still uses PARP inhibitors as maintenance treatment agents. All of these studies utilized platinum-based agents with or without immunotherapy treatment as the first-line therapy.

The MEDIOLA trial (NCT02734004), a phase I/II study, evaluated the combination of olaparib and durvalumab in platinum-sensitive EOC. The first stage of this trial was conducted in women with platinum-sensitive recurrent EOC associated with germline BRCA mutation. The second stage was performed in patients with platinum-sensitive recurrent EOC with or without BRCA mutation. In the phase II study involving 32 patients with germline BRCA1/2 mutant platinum-sensitive ovarian cancer, the disease control rate (DCR) at 12 weeks was 81%, and the objective response rate (ORR) was 63%, with acceptable toxicity (85). Aside from platinum-sensitive recurrent patients, a phase I/II study (TOPACIO, NCT02657889) evaluated the effects of niraparib combined with anti-PD-1 antibody pembrolizumab therapy in platinum-resistant or refractory recurrent ovarian cancer. This therapy strategy was well tolerated and had an ORR of 18% and a clinical benefit rate of 65%, exceeding that of monotherapy of either drug in platinum-resistant recurrent ovarian cancer clearly (86). NCT02484404 is a phase I/II study of durvalumab in combination with olaparib and/or cediranib for advanced or recurrent ovarian cancer. However, the combined durvalumab and olaparib therapy did not show a significant improvement in clinical efficacy according to the RECIST criteria, with an ORR of only 14% irrespective of BRCA status and a DCR of 71%. A third of the patients received clinical benefit lasting longer than 6 months, irrespective of BRCA mutation status in patients with heavily pretreated platinum-resistant recurrent ovarian cancer (87). À correlative analysis of fresh core biopsy and blood samples collected from this trial matching pre- and on-therapy found that the treatment enhanced CXCL9/CXCL10 and IFNγ expression, systemic IFNγ/TNFα production, and TILs, indicating that olaparib/durvalumabhad has a immunomodulatory effect on patients (88). The IFNγ expression correlated positively with the clinical efficacy of this combination therapy, whereas the level of VEGFR3 was negatively associated with PFS, suggesting that VEGF/VEGFR pathway blockade would improve the efficacy of this combination (88). Several clinical trials are under way to investigate the efficacy of PARP inhibitors in combination with ICI and target VEGF drugs in the treatment of ovarian cancer. NCT02734004 and NCT03806049 investigated the efficacy of the treatment of platinum-sensitive EOC. NCT03574779 is a phase II study that aims to evaluate the efficacy and safety of niraparib, TSR-042, and bevacizumab in platinum-resistant ovarian cancer. NCT02873962, a phase II trial, seeks to evaluate the efficacy of the combination of nivolumab, bevacizumab, and rucaparib in the treatment of relapsed ovarian cancer, regardless of the platinum reaction state.

Many studies evaluated the efficacy of various combinations of PARP inhibitors (olaparib, niraparib, rucaparib, and talazoparib) and ICI agents [anti-PD-1 antibodies (pembrolizumab and nivolumab), anti-PD-1 antibodies (avelumab, atezolizumab, and durvalumab(and anti-CTLA-4 antibodies (tremelimumab)] in the treatment of patients with ovarian cancer (**Table 1**). A clinical study indicated that other key mediators of DDR, such as CHK1, ATM, CHK2, and ATR, may also contribute to ICI combinations in several tumor types (89), and the efficacy of other DDR inhibitors combined with ICI in the treatment of ovarian cancer requires further validation.

**Table 1:** Clinical trials using poly (ADP ribose) polymerase (PARP) inhibitor and immunotherapy in ovarian cancer.

Treatment setting	Trial	Agents	Phase	design	Patients/sample size	Primary endpoint
First line	NCT03602859 (FIRST trial)	Drug: niraparib, TSR-042 Chemo + placebo, maintenance placebo Chemo + placebo, maintenance niraparib Chemo + TSR-042, maintenance niraparib + TSR-042	III	Maintenance	Stage III/IV EOC *n* = 912	PFS
	NCT03642132 (JAVELIN Ovarian PARP 100)	Drug: talazoparib, avelumab Chemo + avelumab, maintenance talazoparib + avelumab Chemo, maintenance talazoparib Chemo + bevacizumab, maintenance bevacizumab	III	Maintenance	Untreated advanced OC *n* = 720	PFS
	NCT03522246 (ATHENA)	Drug: rucaparib, nivolumab Rucaparib + nivolumab Rucaparib + placebo Placebo + nivolumab Placebo + placebo	III	Maintenance	Stage III/IV EOC, platinum sensitive *n* = 1,012	PFS
	NCT03737643 (DUO-O)	Drug: olaparib, durvalumab, bevacizumab Olaparib placebo + bevacizumab + durvalumab placebo Olaparib placebo + bevacizumab + durvalumab Olaparib + bevacizumab + durvalumab	III	Maintenance	Newly diagnosed stage III/IV OC *n* = 1,056	PFS
	NCT03740165 (KEYLYNK-001)	Drug: pembrolizumab, olaparib, bevacizumab Chemo, maintenance pembrolizumab + olaparib Chemo, maintenance pembrolizumab + olaparib placebo Chemo, maintenance pembrolizumab placebo + olaparib placebo	III	Maintenance	BRCA non-mutated stage III/IV OC *n* = 1,086	PFS OS
Recurrent (platinum sensitive)	NCT02734004 (MEDIOLA)	Drug: olaparib, bevacizumab, durvalumab Olaparib + durvalumab Olaparib + durvalumab + bevacizumab	I/II	Treatment	First stage: platinum-sensitive EOC, germline BRCA mutated; Second stage: platinum-sensitive EOC with or without BRCA mutation *n* = 427	DCR ORR
	NCT03330405 (JAVELIN PARP Medley trial)	Drug: avelumab, talazoparib Avelumab + talazoprib	Ib/II	Treatment	Platinum-sensitive recurrent EOC, with or without BRCA mutation *n* = 296	DLT OR
	NCT04034927	Drug: olaparib, tremelimumab Olaparib Olaparib + tremelimumab	II	Treatment	Platinum-sensitive OC *n* = 170	PFS DLT
	NCT03806049	Drug: niraparib, TSR-042, bevacizumab Chemo + niraparib + TSR-042 + bevacizumab Chemo + niraparib + bevacizumab Chemo	III	Treatment	Platinum-sensitive EOC *n* = 337	PFS
	NCT03695380	Drug: cobimetinib, niraparib, atezolizumab Cobimetinib + niraparib Cobimetinib + niraparib + atezolizumab	Ib	Treatment	Advanced platinum-sensitive OC *n* = 70	AEs Laboratory test changed ORR
Recurrent (platinum resistant)	NCT02657889 (TOPACOP/Keynote-162)	Drug: niraparib, pembrolizumab Niraparib + pembrolizumab	I/II	Treatment	Recurrent platinum-resistant OC *n* = 114	DLT ORR
	NCT03574779 (OPAL)	Drug: niraparib, TSR-042, bevacizumab Niraparib + TSR-042 + bevacizumab	II	Treatment	Platinum-resistant high-grade EOC *n* = 40	ORR
	NCT03955471 (MOONS TONE)	Drug: niraparib, TSR-042 Niraparib + TSR-042	II	Treatment	Platinum-resistant OC *n* = 150	ORR
Recurrent (independent platinum reaction state)	NCT02571725	Drug: tremelimumab, olaparib Olaparib + tremelimumab	I/II	Treatment	Platinum-sensitive or -resistant recurrent EOC, with germline BRCA1 or BRCA2 mutation *n* = 50	RP2D ORR
	NCT02953457	Drug: olaparib, durvalumab Olaparib + durvalumab Olaparib + tremelimumab	I/II	Treatment	Platinum-sensitive or -resistant EOC, with BRCA1 or BRCA2 germline or somatic mutation *n* = 39	DLT PFS
	NCT03651206 (ROCSAN)	Drug: niraparib, TSR-042 Phase II: niraparib Niraparib + TSR-042 Niraparib + chemotherapy Phase III: the best arm of the phase II Chemotherapy drugs	II/III	Treatment	Metastatic or recurrent OC *n* = 196	RR OS
	NCT03598270 (ANITA)	Drug: atezolizumab, niraparib Chemo + atezolizumab, maintenance niraparib + atezolizumab Chemo + atezolizumab, maintenance niraparib + placebo	III	Maintenance	Recurrent OC *n* = 414	PFS
	NCT02484404	Drug: cediranib, durvalumab, olaparib Cediranib + durvalumab Olaparib + durvalumab Olaparib + durvalumab + cediranib	I/II	Treatment	Recurrent EOC *n* = 384	RP2D ORR
	NCT02873962	Drug: nivolumab, bevacizumab, rucaparib Nivolumab + bevacizumab Nivolumab + bevacizumab + rucaparib	II	Treatment	Recurrent EOC *n* = 76	ORR
	NCT02485990	Drug: olaparib, tremelimumab Tremelimumab Tremelimumab + olaparib	I/II	Treatment	Recurrent or persistent OC *n* = 68	DLT
	NCT03101280	Drug: rucaparib, atezolizumab Rucaparib + atezolizumab	Ib	Treatment	Advanced or metastatic platinum-sensitive OC *n* = 48	AE DLT RP2D
	NCT03824704 (ARIES)	Drug: rucaparib, nivolumab Rucaparib + nivolumab	II	Treatment	Platinum-treated advanced OC *n* = 139	ORR
	NCT04169841 (GUIDE2REPAI)	Drug: durvalumab, tremelimumab, olaparib Durvalumab + tremelimumab + olaparib	II	Treatment	Carriers of HR repair genes mutation in response or stable after olaparib treatment *n* = 270	PFS

EOC, epithelial ovarian cancer; OC, ovarian cancer; ORR, overall response rate; DCR, disease control rate; DLT, incidence of dose-limiting toxicities; OR, overall response; RP2D, recommended phase II dose; RR, response rate; AE, adverse events.

## Potential Predictive Biomarkers for Combination Therapy

A study on PARP inhibitor and anti-PD-L1 combination therapy found no clinical efficacy and no significant changes in TMB or STING expression. Nevertheless, a third of the patients received clinical benefit on platinum-resistant and heavily pretreated ovarian cancer (88). A retrospective pan-cancer analysis revealed that TMB predicted an increased response to ICI in the cancer types where CD8^+^T cell levels correlated with TNB positively (90). However, tumor types where CD8^+^T cell levels did not correlate with TNB showed a significantly lower ORR in TMB-high tumors (90). This result suggested that reliable predictive biomarkers independent of TMB are required to guide the selection of patients who are most likely to benefit from the treatment. The discovery of biomarkers in peripheral blood is helpful in monitoring the effects of combination therapy, especially for patients with heavily pretreated ovarian cancer. Combination therapy induces immune activation probably in a STING-independent manner, including increasing the expression of IFNγ and related immunostimulatory chemokines, enhancing the systemic production of TNFα and IFNγ, and increasing the number of TILs in patients with ovarian cancer. Lampert et al. found that an increase in IFNγ plasma level after treatment is associated with improved response to PFS (88). The overexpression of angiogenic factors in the tumor microenvironment has been preclinically shown to promote immunosuppression and facilitate cancer growth and metastases (91). Lampert et al. also demonstrated that increased post-treatment levels of VEGFR3 are associated with worse PFS in ovarian cancer (88). Immunogenomic profiling analysis on tumor samples from TOPACIO trial showed mutational signature 3 as a surrogate of homologous recombination deficiency (HRD) and a positive immune score reflecting interferon-primed CD8-exhausted effector T-cells in a tumor microenvironment, and both of them were identified as determinants of response to niraparib plus pembrolizumab combination therapy (92).

In high-grade serous ovarian cancer, mutations in synthetic lethality targets for PARP inhibitors can result in high neoantigen load, increased TILs, and enhanced PD-1 and PD-L1 expression. Therefore, mutations in BRCA1/BRCA2 or HRD genes might be effective predictive biomarkers for combination therapy (45). In patients with breast invasive carcinoma, colon adenocarcinoma, and uterine corpus endometrial carcinoma, BER-defective tumors exhibit elevated neoantigen production and upregulated PD-L1 expression (93). However, clinical evidence on the use of combination therapy in BER-defective tumors in ovarian cancer has not been reported. FDA approved microsatellite instability/defective mismatch repair (MSI/dMMR) as a DDR defect biomarker to predict responses to ICI inhibitor therapy (94). dMMR tumors harbor numerous mutations that are associated with T cell infiltration and high neoantigen load. MSI/dMMR is a potential biomarker for combined targeting on the MMR pathway and ICI therapy. In addition, whether other factors that lead to genomic instability, such as NER-defective (95) or POLD1/POLE mutations (96, 97), can act as predictive biomarkers for ovarian cancer needs to be validated further. A single biomarker is insufficient to predict patients who will likely benefit from combination therapy based on our current understanding of clinical response. The discovery of combined predictive biomarkers is of great significance for the selection of benefit subgroups.

## Conclusions and Future Perspectives

The combination of DDR inhibitors and ICI agents is a promising novel modality in cancer treatment. In ovarian cancer, PARP inhibitors were initially designed for BRCA mutations in patients with ovarian cancer. PARP inhibitors were then observed to induce tumor genomic instability and immune modulation, thereby increasing antitumor immune responses. Owing to the modest response of monotherapy in ovarian cancer, the combination therapy of PARP inhibitors with ICI agents provides an opportunity to increase the effectiveness of therapy. In this review, we summarized existing evidence on the relationship between DDR pathways and ICI responses. We summarized current ongoing clinical trials on combinations of PARP inhibitors with ICIs. In to-date clinical trials, combination therapy has achieved good therapeutic effects on ovarian cancer treatment. However, they have not significantly increased the antitumor effects compared with those individual agents.

A next critical step is to identify reliable predictive biomarkers, especially the joint prediction of multiple markers for selecting optimal patient population that will benefit the most from this combination. Current clinical studies on PARP inhibitors combined with ICI for the treatment of ovarian cancer are mainly divided into four indications: first-line maintenance treatment, platinum-sensitive treatment, platinum-resistant treatment and regardless of platinum response state. Selecting specific biomarkers for different indications for patients is particularly important to achieve precision therapy. In addition, it is necessary to determine the standardized criteria and cutoff threshold of biomarkers for the clinical selection of patients. *In vitro* and *in vivo* experiments should be conducted to understand the drug resistance mechanisms of combination drugs to achieve the role of an early warning system. Another method for increasing the efficacy of combination therapy is by converting nonresponsive “cold tumor” into responsive “hot” tumors. The pharmacological activation of cGAS–STING signaling pathway is also under investigation (39). ADU-S100 and DMXAA are STING agonists that promote type I IFN induction and CD8+ T cell activation to increase antitumor responses. In this regard, addition of drugs, such as STING agonists or VEGF/VEFGR pathway blockade, which can modulate the immunosuppressive microenvironment in ovarian cancer may be necessary to improve the efficacy of PARP inhibitor and ICI combination therapy. This avenue for research warrants further investigation.

Given that DDR deficiency plays a key role in immunotherapy response, other targeted agents that move beyond PARP in targeting DDR pathways, such as CHK1, CHK2, ATM, and ATR inhibitors, may also contribute to ICI combination therapy for ovarian cancer. The schedule and optimal dose for combination treatment needs to be further determined. The safety of combination therapy needs to be evaluated by clinical trials. In addition, the selection of biomarkers utilized to screen the benefit to patients is crucial to achieve precise treatment. Finally, it is essential to have a comprehensive understanding of immune responses to DNA damage at the cellular and organismal levels. Such an understanding can help in identifying potentially novel targets for future cancer treatments.

## Author Contributions

BX and WJ designed the manuscript. HX and WW wrote the manuscript. HX and WQ drew the figures and tables. WJ and BX revised the manuscript. All authors contributed to the article and approved the submitted version.

## Funding

This work was supported by the National Natural Science Foundation of China (grant number 82003551), the China Postdoctoral Science Foundation (grant numbers 2019M661309 and 2019M661304), and the Heilongjiang Province Postdoctoral Science Foundation (grant number LBH-Z19081).

## Conflict of Interest

The authors declare that the research was conducted in the absence of any commercial or financial relationships that could be construed as a potential conflict of interest.

## Publisher’s Note

All claims expressed in this article are solely those of the authors and do not necessarily represent those of their affiliated organizations, or those of the publisher, the editors and the reviewers. Any product that may be evaluated in this article, or claim that may be made by its manufacturer, is not guaranteed or endorsed by the publisher.
